# Surfactin Containing *Bacillus licheniformis*-Fermented Products Alleviate Dextran Sulfate Sodium-Induced Colitis by Inhibiting Colonic Inflammation and the NLRP3 Inflammasome in Mice

**DOI:** 10.3390/ani12243456

**Published:** 2022-12-07

**Authors:** Wei-Che Tsai, Wei-Ting Wong, Hsien-Ta Hsu, Yeong-Hsiang Cheng, Yu-Hsiang Yu, Wei-Jung Chen, Chen-Lung Ho, Hui-Chen Hsu, Kuo-Feng Hua

**Affiliations:** 1 Division of Cardiology, Department of Internal Medicine, Tri-Service General Hospital, National Defense Medical Center, Taipei 114202, Taiwan; 2 Department of Biotechnology and Animal Science, National Ilan University, Yilan 260007, Taiwan; 3 Division of Neurosurgery, Taipei Tzu Chi Hospital, Buddhist Tzu Chi Medical Foundation, New Taipei City 231405, Taiwan; 4 School of Medicine, Buddhist Tzu Chi University, Hualien 97004, Taiwan; 5 Division of Wood Cellulose, Taiwan Forestry Research Institute, Taipei 100051, Taiwan; 6 Department of Medical Research, China Medical University Hospital, China Medical University, Taichung 404333, Taiwan

**Keywords:** *Bacillus licheniformis*, surfactin, inflammatory bowel disease, colitis, NLRP3 inflammasome

## Abstract

**Simple Summary:**

Quality of life is significantly impaired among individuals with inflammatory bowel disease (IBD), and the growth performance of animals is also affected by IBD. However, the side effects and poor therapeutic effects of medications for IBD limit the treatment effect. In this study, we demonstrated that oral administration of surfactin containing *Bacillus licheniformis*-fermented products (SBLF) alleviates dextran sulfate sodium-induced colitis in mice by reducing inflammatory responses and the NLRP3 inflammasome activation in colon tissue.

**Abstract:**

Inflammatory bowel disease (IBD) is a non-infectious disease characterized by chronic inflammation of the gastrointestinal tract. Currently, management of IBD is still a clinical challenge. The purpose of this study was to investigate the therapeutic potential of surfactin containing *Bacillus licheniformis*-fermented products (SBLF) and commercial surfactin (CS) on the treatment of dextran sulfate sodium (DSS)-induced colitis in a mouse model. We found that mice that received drinking water containing 3% DSS developed significant colitis symptoms, including increased disease activity index, body weight loss, shortening of the colon length, splenomegaly, colonic inflammation and colonic NOD-, LRR- and pyrin domain-containing protein 3 (NLRP3) inflammasome activation. Notably, orally received SBLF, CS or clinical anti-inflammatory drug 5-aminosalicylic acid improved DSS-induced colitis symptoms in mice. These findings show that SBLF can improve IBD in mice by reducing colonic inflammation and inhibiting the NLRP3 inflammasome activation, suggesting that SBLF has the potential to be used as a nutraceutical in humans or a feed additive in economic and companion animals for preventing IBD.

## 1. Introduction

The gut is the largest immune organ in the body and most immune cells can be found in the gut. The gut uses mucus and epithelial cells as a physical barrier to keep approximately 100 trillion microorganisms in the gastrointestinal tract [1]. However, when this physical barrier of the gut is compromised, immune cells will overreact to invading microorganisms, food antigens or self-antigens, resulting in intestinal inflammation, which may lead to inflammatory bowel disease (IBD) [2]. The two most common forms of IBD are Crohn’s disease and ulcerative colitis, it is characterized by chronic inflammation of the gastrointestinal tract, and affects more than 6.8 million people worldwide [3]. IBD does not only affect humans, for example, dogs and cats are also susceptible to inflammatory bowel diseases similar to humans, such as lymphocytic/plasmacytic enteritis and eosinophilic gastroenteritis [4]. Recently, it has been reported that coinfection with porcine epidemic diarrhea virus and bovine viral diarrhea virus may lead to IBD in piglets [5]. Therefore, IBD is a problem that veterinary medicine needs to resolve in economic and companion animals; however, this is challenging due to the difficulty of diagnosis and the lack of effective treatment and prevention methods.

The treatment of IBD mainly relies on anti-inflammatory drugs (e.g., 5-aminosalicylic acid, 5-ASA), immunosuppressants and antibiotics [6]. These treatments have many problems, mainly including side effects and poor therapeutic effects [7]. Therefore, the development of novel therapies for IBD is urgent. The intracellular sensor NOD-, LRR- and pyrin domain-containing protein 3 (NLRP3) inflammasome is a protein complex containing NLRP3, apoptosis-associated speck-like protein containing a CARD (ASC) and caspase-1, which regulates the activity of the protease caspase-1 that processes the precursors of interleukin (IL)-1β and IL-18 in many cells [8]. NLRP3 inflammasome plays an important pathogenic role in IBD [9]. In the experimental model of IBD in mice, dextran sodium sulfate (DSS) is the most commonly used agent to induce IBD and activate the NLPP3 inflammasome [10]. Targeting the NLPP3 inflammasome is a promising therapeutic strategy for IBD [11].

*Bacillus licheniformis* is a facultative anaerobic Gram-positive bacterium that exists in soil or plants and forms endospores for its survival in difficult environments. Surfactin is a powerful lipopeptide biosurfactant that can be produced by *B. licheniformis* [12]. We have established a solid-state fermentation method for *B. licheniformis* and demonstrated that the *B. licheniformis*-fermented products contain 5 × 10^9^ CFU/g *B. licheniformis* spore and 14.3 mg/g surfactin, respectively [13]. The surfactin-containing *B. licheniformis*-ferments (SBLF) has been shown to exert antiviral activity against the porcine epidemic diarrhea virus [14]. However, the effect of SBLF on IBD is unexplored. Here, we investigate the therapeutic potential of SBLF on IBD in a mouse model of DSS-induced colitis.

## 2. Materials and Methods

### 2.1. Materials

Commercial surfactin (CS) from *Bacillus subtilis* was obtained from Sigma-Aldrich (St. Louis, MO, USA). DSS (M.W. 36,000–50,000 Da) was obtained from MP Biomedicals (Irvine, CA, USA). ELISA kits for IL-1β, IL-6, tumor necrosis factor-α (TNF-α) and monocyte chemoattractant protein-1 (MPC-1) were obtained from Thermo Fisher Scientific (Waltham, MA, USA). NLRP3 antibody was obtained from Adipogen Life Science (SanDiego, CA, USA). IL-1β antibody was obtained from R&D Systems (Minneapolis, MN, USA). Antibodies against Actin, ASC, cyclooxygenase-2 (COX-2) and 5-ASA were obtained from Santa Cruz Biotechnology (Santa Cruz, CA, USA). A myeloperoxidase (MPO) activity assay kit was obtained from Abcam (Cambridge, UK).

### 2.2. Preparation of SBLF

*B. licheniformis* (ATCC 12713) was purchased from the Food Industry Research and Development Institute (Hsinchu, Taiwan). The method used for SBLF preparation was described in our previous study [13]. Briefly, *B. licheniformis* were inoculated in sterile wheat bran and soybean meal-based substrates and fermented for 6 days at 30 °C in an anaerobic chamber with relative humidity above 80%. After fermentation, the solid-state fermented products were dried by incubation at 50 °C for 2 days. The dried fermented products were ground into a fine powder using a grinder to obtain SBLF.

### 2.3. Mouse Model of DSS-Induced Colitis

C57BL/6JNal male mice (six-weeks old) were obtained from the National Laboratory Animal Center (Taipei, Taiwan), and housed in the National Ilan University Animal Center at room temperature (23 ± 3 °C) and relative humidity (40–60%). The research design followed previous studies with minor modifications [15,16]. The mice were randomized into 7 groups: H_2_O + vehicle group, received H_2_O with oral gavage of vehicle (0.5% methanol) daily, n = 3; DSS + vehicle group, received 3% (wt/vol) DSS in H_2_O with oral gavage of vehicle daily, n = 6; DSS + CS group, received 3% DSS in H_2_O with oral gavage of 20 mg/kg CS daily, n = 6; DSS + 20 mg/kg SBLF group, received 3% DSS in H_2_O with oral gavage of 20 mg/kg SBLF daily, n = 6; DSS + 5-ASA group, received 3% DSS in H_2_O with oral gavage of 40 mg/kg 5-ASA daily, n = 6; H_2_O + CS group, received H_2_O with oral gavage of 20 mg/kg CS daily, n = 3; H_2_O + SBLF group, received H_2_O with oral gavage of 20 mg/kg SBLF daily, n = 3. For DSS treatment, mice received 3% DSS in H_2_O and normal diet ad libitum for 5 days, followed by H_2_O and starvation for 1 day. CS, SBLF or 5-ASA were given daily by oral gavage during the DSS treatment. The body weight and disease activity index (DAI) of each mouse were recorded daily. On Day 7, following induction with DSS, mice were sacrificed for the collection of colonic tissue and spleen.

### 2.4. DAI Scoring

The DAI score is a research tool used to quantify the symptoms of mice with IBD. The DAI score was calculated by combining the stool score and bleeding score. The stool scores included: 0 = well-formed stools; 1 = semi-formed stools that did not adhere to the anus; 2 = semi-formed stools that adhered to the anus. The bleeding scores included: 0 = no bleeding; 1 = slight bleeding; 2 = gross bleeding.

### 2.5. Analysis of the Levels of Cytokine, Chemokine and MPO in Colons and Serum

The colonic tissues from mice were homogenized with lysis buffer followed by centrifugation at 13,000 rpm at 4 °C for 20 min. The protein concentration was determined by the BCA protein assay. The expression levels of IL-1β, IL-6, TNF-α, and MPC-1 in the colonic tissues were analyzed by ELISA. The expression levels of IL-1β in serum were analyzed by ELISA. The activity of MPO in colonic tissues was analyzed by the colorimetric activity assay kit.

### 2.6. H&E Analysis and Histopathological Scoring

Colonic tissues were fixed in 10% formalin and embedded in paraffin. Sections were stained with H&E by Energenesis Biomedical Co., Ltd. (Taipei, Taiwan). Histopathological scores were calculated by a pathologist by combining the inflammatory cell infiltration score and tissue damage score as described previously [15]. The inflammatory cell infiltration scores were as follows: 0 = lamina propria with rare inflammatory cells; 1 = lamina propria with some inflammatory cells; 2 = confluence of inflammatory cells extending into the submucosa; 3 = transmural extension of the infiltrate. The tissue damage scores were: 0 = no damage; 1 = lymphoepithelial lesions; 2 = surface mucosal erosion or focal ulceration; 3 = extensive mucosal damage and extension into deeper structures of the bowel wall.

### 2.7. Statistical Analysis

The two-tailed t test was used to analyze two groups. For the analysis of three or more groups, ANOVA with Dunnett’s multiple comparisons test was used. The standard deviation of separate experiments is indicated by error bars. *, ** and *** represent *p* < 0.05, *p* < 0.01. and *p* < 0.001, respectively.

## 3. Results

### 3.1. SBLF Ameliorates DSS-Induced Diarrhea and Bloody Stool and Improves Body Weight Loss in a Mouse Model

**Figure 1:**
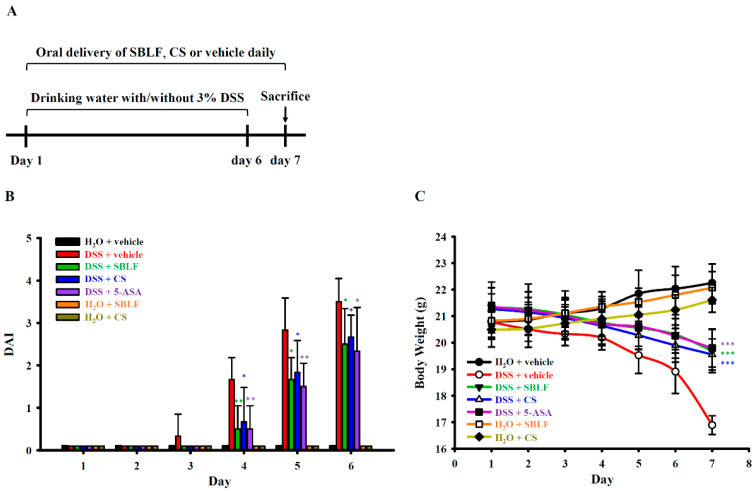
SBLF improves DSS-induced colitis in mice. (**A**) Experimental design of mouse model. (**B**) Effect of SBLF on DAI. (**C**) Effect of SBLF on body weight loss. Bars and lines show the mean ± SD for separate experiments. * *p* < 0.05, ** *p* < 0.01 and *** *p* < 0.001 compared to vehicle + DSS group.

To investigate whether SBLF can improve inflammatory bowel disease, the effect of SBLF on colitis in DSS-treated mice was investigated (Figure 1A). Mice that received drinking water containing 3% DSS developed significant diarrhea and bloody stool on day 5 post DSS administration and until they were sacrificed on day 7. Mice that orally received SBLF or CS had significantly reduced DSS-induced diarrhea and bloody stool. Importantly, mice that orally received SBLF or CS alone did not have diarrhea and bloody stool. The DAI scores of mice were calculated based on the severity of the diarrhea and bloody stool. We found that DSS induced a DAI score of 1.67 ± 0.52, 2.83 ± 0.75 and 3.50 ± 0.55 on day 4, day 5 and day 6 post-DSS administration, respectively (Figure 1B). DSS-treated mice that orally received SBLF showed a significantly reduced DAI score of 0.5 ± 0.55 (*p* < 0.01), 1.67 ± 0.52 (*p* < 0.05) and 2.50 ± 0.84 (*p* < 0.05) on day 4, day 5 and day 6 post-DSS administration, respectively (Figure 1B). Similar to the effect of SBLF, DSS-treated mice that orally received CS showed a significantly reduced DAI score of 0.67 ± 0.82 (*p* < 0.05), 1.83 ± 0.75 (*p* < 0.05) and 2.67 ± 0.52 (*p* < 0.05) on day 4, day 5 and day 6 post-DSS administration, respectively (Figure 1B). As expected, DSS-treated mice that orally received 5-ASA showed a significantly reduced DAI score of 0.50 ± 0.55 (*p* < 0.05), 1.50 ± 0.55 (*p* < 0.05) and 2.33 ± 1.03 (*p* < 0.05) on day 4, day 5 and day 6 post-DSS administration, respectively (Figure 1B). In addition, body weight loss is one of the major symptoms of DSS-induced colitis in mice. The body weight of mice that received H_2_O increased to 106.94 ± 3.22%, while in mice that received DSS, it decreased to 81.28 ± 2.10% on day 7 compared to day 1 (Figure 1C). In mice that received DSS with SBLF, CS or 5-ASA, it decreased to 92.28 ± 4.14% (*p* < 0.001 compared to DSS), 91.95 ± 3.01% (*p* < 0.001 compared to DSS) or 92.73 ± 3.63% (*p* < 0.001 compared to DSS) on day 7 compared to day 1 (Figure 1C). These results indicated that both SBLF and CS can improve DSS-induced colitis in mice.

### 3.2. SBLF Ameliorates DSS-Induced Colonic Damage in Mice

**Figure 2:**
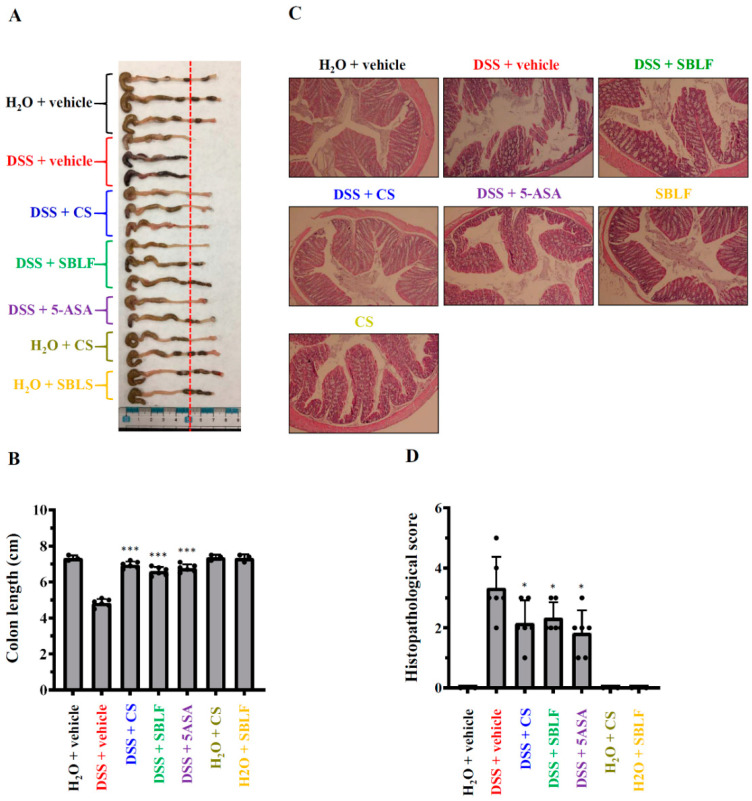
SBLF improves colon damage in DSS-treated mice. (**A**) Images of representative colon from mice. (**B**) Bars represent the colon length of mice. (**C**) Images of representative H&E stained colon sections from mice. (**D**) Histopathological score for the colon from mice. Bars show the mean ± SD. * *p* < 0.05 and *** *p* < 0.001 compared to vehicle + DSS group.

As SBLF ameliorates diarrhea in DSS-treated mice, we further investigated whether SBLF ameliorates colonic damage. The colon length of mice that received H_2_O was 7.33 ± 0.15 cm, while the colon length of mice that received drinking water containing 3% DSS decreased to 4.83 ± 0.22 cm (Figure 2A,B). SBLF, CS and 5-ASA significantly reduced colonic shortening in DSS-treated mice by increased the colon length to 6.60 ± 0.24 cm (*p* < 0.001 compared to DSS), 6.95 ± 0.19 cm (*p* < 0.001 compared to DSS) and 6.77 ± 0.22 cm (*p* < 0.001 compared to DSS), respectively (Figure 2A,B). Mice that orally received SBLF or CS alone did not exhibit colonic shortening compared to the mice that received H_2_O (Figure 2A,B). In addition, the severity of colitis in mice was further evaluated by histopathological analysis using H&E staining. The colon tissues from mice that received H_2_O showed no infiltration of the inflammatory cells, no lymphoepithelial lesions, and intact mucosa and bowel wall (Figure 2C,D). The colon tissues from mice that received drinking water containing 3% DSS, exhibited severe damage in the surface epithelium and mucosa and infiltration of the inflammatory cells (Figure 2C,D). SBLF, CS and 5-ASA significantly attenuated DSS-induced colonic damage by reducing the histopathological score (Figure 2C,D). These results indicated that SBLF and CS improves colonic damage in DSS-treated mice.

### 3.3. SBLF Ameliorates DSS-Induced Splenomegaly and Colonic Inflammation in Mice

**Figure 3:**
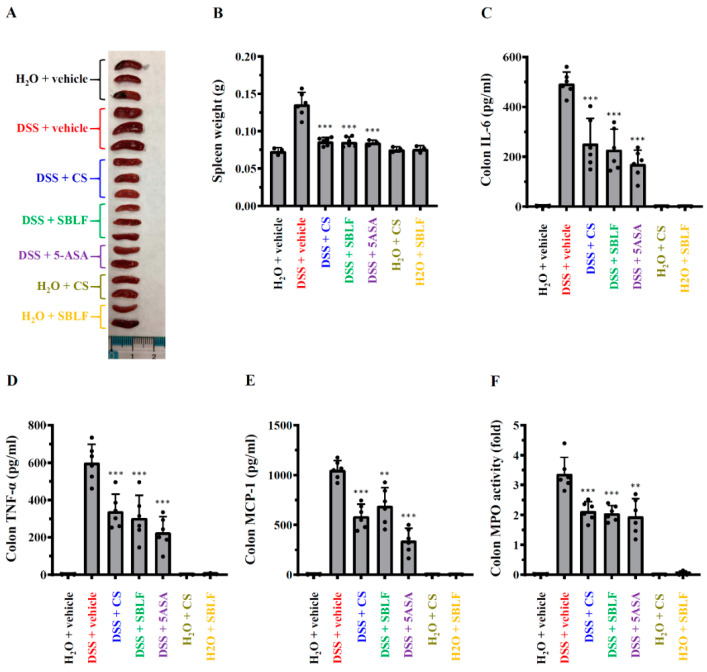
SBLF improves inflammation in DSS-treated mice. (**A**) Images of representative splenomegaly of mice. (**B**) Bars represent the spleen weight of mice. (**C**) The levels of IL-6 in colon tissue. (**D**) TNF-α concentration in colon tissue. (**E**) MCP-1 concentration in colon tissue. (**F**) MPO activity in colon tissue. Bars show the mean ± SD. ** *p* < 0.01 and *** *p* < 0.001 compared to vehicle + DSS group.

Mice that received H_2_O containing 3% DSS exhibited significant splenomegaly. The weight of spleen from DSS-treated mice was 0.135 ± 0.016 g, while it was 0.073 ± 0.005 g from mice that received normal drinking water (Figure 3A,B). SBLF, CS and 5-ASA significantly attenuated DSS-induced splenomegaly by reducing the spleen weight to 0.086 ± 0.007 g (*p* < 0.001 compared to DSS), 0.086 ± 0.005 g (*p* < 0.001 compared to DSS) and 0.085 ± 0.004 g (*p* < 0.001 compared to DSS), respectively (Figure 3A,B). In addition, there was no detectable IL-6, TNF-α, MCP-1 and MPO in the colon tissue from mice that received normal drinking water. The levels of IL-6 in colon tissue from DSS-treated mice increased to 494.1 ± 45.7 pg/mL, and this effect was reduced by SBLF (228.4 ± 82.0 pg/mL, *p* < 0.001 compared to DSS), CS (253.4 ± 99.9 pg/mL, *p* < 0.001 compared to DSS) and 5-ASA (171.3 ± 55.2 pg/mL, *p* < 0.001 compared to DSS) (Figure 3C). DSS increased TNF-α expression in the colon tissue (599.7 ± 97.8 pg/mL), and the levels of TNF-α were reduced to 304.3 ± 119.8 pg/mL (*p* < 0.001 compared to DSS), 339.8 ± 91.3 pg/mL (*p* < 0.001 compared to DSS) and 226.3 ± 84.0 pg/mL (*p* < 0.001 compared to DSS) by SBLF, CS and 5-ASA, respectively (Figure 3D). The level of MCP-1 in colon tissue from DSS-treated mice increased to 1052.2 ± 92.5 pg/mL, while it was reduced by SBLF (691.8 ± 180.7 pg/mL, *p* < 0.01 compared to DSS), CS (588.1 ± 119.2 pg/mL, *p* < 0.001 compared to DSS) and 5-ASA (342.3 ± 124.7 pg/mL, *p* < 0.001 compared to DSS) (Figure 3E). In addition, in mice that received drinking water containing 3% DSS, the activity of MPO in colon tissue (3.37 ± 0.55 folds) also increased compared to mice that received drinking water, and this effect was reduced by SBLF (2.05 ± 0.26 folds, *p* < 0.001 compared to DSS), CS (2.13 ± 0.32 folds, *p* < 0.001 compared to DSS) and 5-ASA (1.95 ± 0.59 folds, *p* < 0.01 compared to DSS) (Figure 3F). These results indicated that SBLF and CS ameliorated DSS-induced colonic inflammation in mice.

### 3.4. SBLF Ameliorates DSS-Induced NLRP3 Inflammasome Activation in Mice

**Figure 4:**
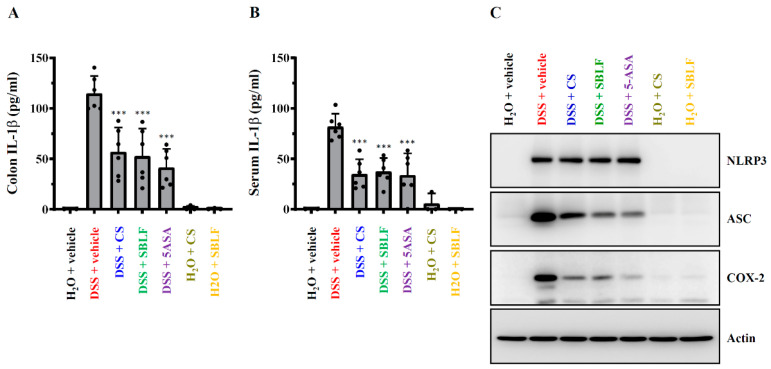
SBLF ameliorates NLRP3 inflammasome activation in DSS-treated mice. (**A**) IL-1β concentration in colon tissue. (**B**) IL-1β concentration in serum. (**C**) The Western blot image represents the protein expression levels in colon tissue. Bars show the mean ± SD. *** *p* < 0.001 compared to vehicle + DSS group.

The levels of IL-1β in colon tissue (Figure 4A) and in serum (Figure 4B) from mice that received drinking water containing 3% DSS increased to 114.93 ± 17.09 pg/mL and 82.04 ± 12.71 pg/mL, respectively, while there was no detectable IL-1β in colon tissue and in serum from mice that received normal drinking water or received SBLF and CS alone. SBLF reduced DSS-induced IL-1β expression in colon tissue and in serum to 52.91 ± 26.90 pg/mL (*p* < 0.001 compared to DSS) and 37.65 ± 13.09 pg/mL (*p* < 0.001 compared to DSS), respectively (Figure 4A,B). CS also reduced DSS-induced IL-1β expression in colon tissue and in serum to 57.04 ± 23.96 pg/mL (*p* < 0.001 compared to DSS) and 34.93 ± 14.70 pg/mL (*p* < 0.001 compared to DSS), respectively (Figure 4A,B). As expected, 5-ASA reduced DSS-induced IL-1β expression in colon tissue and in serum to 41.42 ± 18.36 pg/mL (*p* < 0.001 compared to DSS) and 33.87 ± 21.53 pg/mL (*p* < 0.001 compared to DSS), respectively (Figure 4A,B). In addition, NLRP3, ASC and COX-2 expression in the colon tissue was increased by DSS. DSS-induced expression of ASC and COX-2 was reduced by SBLF, CS and 5-ASA; however, NLRP3 expression was not affected (Figure 4C). The induction of NLRP3 and the IL-1β precursor from priming signals activated by pathogen-associated molecular patterns (e.g., LPS) is insufficient to induce the activation of the NLRP3 inflammasome, and a second stimulus from activation signals activated by damage-associated molecular patterns (e.g., ATP) is needed [8]. Here, we demonstrated that SBLF significantly reduced the NLRP3 inflammasome’s end product, IL-1β expression in colon tissues; however, NLRP3 expression was not affected. These findings showed that SBLF reduced NLRP3 inflammasome activation through inhibiting activation signals, but not priming signals.

## 4. Discussion

IBD is a global disease associated with a low quality of life that affects more than 6.8 million people worldwide [3]. Although the pathogenesis of IBD is complicated and remains largely unclear, it is believed that abnormal intestinal immune responses caused by genetic, environmental or microbial factors play an important role in the disease development [17]. Medications are the first step in the treatment of IBD, such as corticosteroids, aminosalicylates, antibiotics, immunosuppressive drugs and supportive drugs, which are commonly used in the first-line treatment of IBD [6]. However, the possible side effects of these medications limit the treatment of IBD. In recent years, reduced microbial diversity has been associated with IBD. The modulation and improvement of gut microbiota by probiotics or other agents has become a potential strategy to prevent or treat IBD [18]. *B. licheniformis* improves colitis and modulates the gut microbiota in DSS-treated mice [19]. In addition, oral administration of *B. subtilis* PB6 suppresses 2,4,6-trinitrobenzene sulfonic acid-induced colitis in rat by reducing pro-inflammatory cytokines and increasing anti-inflammatory cytokines in the plasma [20]. The phospholipase A2, a rate-limiting enzyme involved in the arachidonic acid associated inflammatory pathway, which is inhibited by surfactins secreted from *B. subtilis* PB6, may be responsible for the protective effect in colitis [20].

Surfactin is a cyclic lipopeptide that is naturally produced by Bacillus genus as a secondary metabolite and it is characterized as one of the most potent biosurfactants [21]. Structurally, surfactin is composed of lipid and protein, with a lipophilic C13-15 β-hydroxy fatty acid side chain linked to a hydrophilic heptapeptide ring [22]. *B. subtilis* is the most common bacterial strain to produce surfactin. In recent years, an increasing number of studies have indicated that *B. licheniformis* is an alternative strain for the anaerobic production of surfactin [12]. In our previous studies, we demonstrated that solid-phase fermentation of *B. licheniformis* using wheat-bran-based solid substrate can produce bioactive surfactin. We found that the SBLF improves the health and growth performance of broilers and pigs by modulating the microbiota [13,14]. Recent studies show that surfactin exerts multiple functions, including anti-bacterial activity [23], anti-cancer activity [24] and anti-inflammatory activity [25]. Based on HPLC analysis, SBLF contains around 1.43% surfactin. We believe that the effect of SBLF on DSS-induced colitis in mice is not only derived from surfactin but also from other unknown components.

DSS-induced colitis is associated with NLPP3 inflammasome activation and excessive IL-1β production [10]. The IL-1β receptor antagonist and caspase-1 inhibitor improved IBD in mice [26]. In addition, inhibition of the NLRP3 inflammasome by MCC950 or glyburide suppressed IBD in mice, confirming the importance of the NLPP3 inflammasome in IBD development [27,28]. Targeting the NLRP3 inflammasome is one of the most important mechanisms of action of drugs that improve IBD [29]. Because food enters the digestive system first, diet is thought to influence the prevalence of IBD, making it a potential adjunctive therapy in IBD [30]. Recently, we demonstrated that *Litsea cubeba* leaves ameliorate colitis in DSS-treated mice by inhibiting the NLRP3 inflammasome [31]. Other studies have also indicated that natural products or dietary supplements have the potential to improve IBD by inhibiting the NLRP3 inflammasome. For example, cardamonin, a natural flavone isolate from *Alpinia katsumadai* Hayata [32], dehydroepiandrosterone, a popular dietary supplement [33], and palmatine, an isoquinoline alkaloid isolated from the traditional herb *Fibraurea Recisa* Pierre [34] attenuated IBD by inhibiting the NLRP3 inflammasome in mice. Here, we demonstrated that SBLF reduced IL-1β levels in colon and serum as well as reducing ASC expression in colon, indicating that SBLF inhibited the NLRP3 inflammasome in DSS-treated mice. However, the detailed mechanism for SBLF-mediated colitis improvement and the NLRP3 inflammasome inhibition was unclear. In our previous study we demonstrated that COX-2 positively regulates NLRP3 inflammasome activation [35]. Here, we demonstrated that SBLF reduced COX-2 expression in colon tissue, which may partially explain how SBLF inhibits the NLRP3 inflammasome in mice. In addition, surfactins from *Bacillus subtilis* have been demonstrated to inhibit phospholipase A2 [20], an enzyme that promotes NLRP3 inflammasome activation in macrophages [36]. These findings suggest that SBLF-mediated NLRP3 inflammasome inhibition may be partially due to phospholipase A2 inhibition.

Both NLRP3 inflammasome and traditional inflammation can be regulated by surfactin. Surfactin significantly inhibited the expression of pro-inflammatory cytokines and mediators, and increased anti-inflammatory cytokine IL-10 expression in copper sulfate-stimulated zebrafish larvae [37]. Surfactin inhibited the expression of pro-inflammatory cytokines and mediators in the epidermis of *Propionibacterium acnes*-infected mice [38]. Surfactin also inhibited COX-2-dependent PGE_2_ production in particulate matter-stimulated human gingival fibroblasts [25]. In this study, we found that SBLF reduced pro-inflammatory cytokines IL-6, TNF-α, MCP-1, MPO and COX-2 in the colon of DSS-treated mice. These results indicated that SBLF alleviates DSS-induced colitis through reducing traditional inflammation in the colon. NF-κB is an important transcription factor in the regulation of proinflammatory cytokines’ expression. NF-κB pathway is activated in colon tissues of mice treated with DSS, as evidenced by increased phosphorylation levels of IκB and IKK and NF-κB p65, indicating that the excessive activation of the NF-κB pathway is one of the most important causes of IBD [39]. Although we demonstrated that SBLF significantly reduced proinflammatory cytokines expression in colon tissue in DSS-treated mice, one limitation of this study is that we were not able to provide solid evidence showing the effect of SBLF on NF-κB activation. This is very important for clarifying the pharmacological mechanism of SBLF in treating IBD in the future.

The intestinal mucosal barrier is formed by the tight junctions of epithelial cells, which is the physical barrier that prevents gastrointestinal tract pathogens from crossing the epithelium [40]. Dysfunction of the intestinal mucosal barrier promotes the development of IBD [41]. Occludin, claudin-1 and zonula occludens-1 are the important tight junction proteins that maintain the integrity of intestinal mucosal barrier. It has been demonstrated that colonic protein levels of occludin, claudin-1 and zonula occludens-1 were down-regulated in DSS-treated mice [42]. Interestingly, a recent study showed that IL-1β increased the permeability of intestinal tight junctions and enhanced intestinal inflammation [43]. We demonstrated that SBLF ameliorated DSS-induced colitis in mice by reducing colonic IL-1β expression and inflammation. It is worth investigating the effect of SBLF on tight junction proteins’ expression and the integrity of intestinal mucosal barrier in the future.

Oxidative stress is characterized by over-production of reactive oxygen species, such as superoxide, hydroxyl radicals and hydrogen peroxide, which may lead to the damage of intracellular components, including proteins, lipids, and nucleic acids etc. [44]. Antioxidant is one of the therapeutic strategies used to treat IBD, indicating that oxidative stress plays a crucial role in IBD development [45]. Under normal physiological conditions, oxidative stress can be controlled through reducing the reactive oxygen species levels by intracellular antioxidant enzymes, such as superoxide dismutase 2, glutathione peroxidase and catalase. The antioxidant enzymes are expressed in the colon tissues of mice, and their expression levels are reduced after DSS treatment [46]. Restoring the expression of antioxidant enzymes in DSS-treated mice is one possible mode-of-action of substances that improve IBD [46]. Although surfactin could induce apoptosis in human oral squamous carcinoma cells through ROS induction [47], it reduced particulate matter-induced ROS production through up-regulation of HO-1 expression in human gingival fibroblasts [48]. The effect of SBLF on DSS-induced oxidative stress in mice needs further investigation.

Paratuberculosis (also known as Johne’s disease) is an infectious disease caused by *Mycobacterium avium* subspecies *paratuberculosis* that primarily affects but is not limited to ruminants. Animals with Johne’s disease exhibit thickening of the intestines, causing reduced nutrient absorption and milk yields, long-lasting diarrhea and body weight loss, and it may affect fertility and mortality [49]. *Mycobacterium avium* subspecies *paratuberculosis* infection is not only a major concern that causes a huge financial burden in livestock farming, but it also attracts public health concerns because this bacterium may cause Crohn’s disease, a human inflammatory bowel disease [50]. Here, we demonstrated that SBLF can improve DSS-induced colitis in mice, suggesting that SBLF has the potential to be used as a food additive for improving Crohn’s disease in ruminants.

In addition, although foodborne illness caused by *B. licheniformis* toxins has been reported, it has only happened in cases with inadequate food preparation conditions. In general, *B. licheniformis* is a safe bacterial strain and non-pathogenic to human and animals [51]. The limitations of this study are that although the anti-colitis effect of SBLF was demonstrated, its long-term safety in mice should be investigated in the future. Another limitation is the lack of cell models to further dissect the molecular mechanism of SBLF-mediated NLRP3 inflammasome inhibition and anti-inflammatory effects. Taken together, our findings suggest that SBLF has the potential to be used as a nutraceutical in humans or a feed additive in economic and companion animals for preventing IBD in the future.

## 5. Conclusions

We demonstrated that SBLF is a natural substance that can alleviate DSS-induced colitis in mice. The beneficial effects of SBLF on colitis are mainly derived from its anti-inflammatory and anti-NLRP3 inflammasome effects. SBLF has the potential to be utilized as a nutraceutical or a feed additive for preventing IBD in humans or economic and companion animals.

## Data Availability

The data presented in this study are available on request from the corresponding author.
